# Ad35 and Ad26 Vaccine Vectors Induce Potent and Cross-Reactive Antibody and T-Cell Responses to Multiple Filovirus Species

**DOI:** 10.1371/journal.pone.0044115

**Published:** 2012-12-06

**Authors:** Roland Zahn, Gert Gillisen, Anna Roos, Marina Koning, Esmeralda van der Helm, Dirk Spek, Mo Weijtens, Maria Grazia Pau, Katarina Radošević, Gerrit Jan Weverling, Jerome Custers, Jort Vellinga, Hanneke Schuitemaker, Jaap Goudsmit, Ariane Rodríguez

**Affiliations:** Crucell Holland BV, Leiden, The Netherlands; Duke-NUS Graduate Medical School, Singapore

## Abstract

Filoviruses cause sporadic but highly lethal outbreaks of hemorrhagic fever in Africa in the human population. Currently, no drug or vaccine is available for treatment or prevention. A previous study with a vaccine candidate based on the low seroprevalent adenoviruses 26 and 35 (Ad26 and Ad35) was shown to provide protection against homologous Ebola Zaire challenge in non human primates (NHP) if applied in a prime-boost regimen. Here we have aimed to expand this principle to construct and evaluate Ad26 and Ad35 vectors for development of a vaccine to provide universal filovirus protection against all highly lethal strains that have caused major outbreaks in the past. We have therefore performed a phylogenetic analysis of filovirus glycoproteins to select the glycoproteins from two Ebola species (Ebola Zaire and Ebola Sudan/Gulu,), two Marburg strains (Marburg Angola and Marburg Ravn) and added the more distant non-lethal Ebola Ivory Coast species for broadest coverage. Ad26 and Ad35 vectors expressing these five filovirus glycoproteins were evaluated to induce a potent cellular and humoral immune response in mice. All adenoviral vectors induced a humoral immune response after single vaccination in a dose dependent manner that was cross-reactive within the Ebola and Marburg lineages. In addition, both strain-specific as well as cross-reactive T cell responses could be detected. A heterologous Ad26–Ad35 prime-boost regime enhanced mainly the humoral and to a lower extend the cellular immune response against the transgene. Combination of the five selected filovirus glycoproteins in one multivalent vaccine potentially elicits protective immunity in man against all major filovirus strains that have caused lethal outbreaks in the last 20 years.

## Introduction

Unpredictable reoccurring sporadic outbreaks of lethal filovirus associated hemorrhagic fever pose a major risk in sub Saharan Africa as they have a high human case fatality rate of 25–90% [1]. Currently no treatment or vaccine is available. Filoviruses that can infect humans and non-human primates are nonsegmented, single-stranded negative- sense RNA viruses that have an unusual filamentous morphology. Filoviridae can be divided into two genera, Ebolavirus and Marburgvirus. Ebola viruses can be further subdivided into five species (Zaire, Sudan, Ivory Coast, Bundibugyo and a non-human primate pathogenic species Reston) whereas the Marburgviruses consist of only one species (Lake Victoria). The human outbreaks are assumed to occur by zoonotic transmission events [2], [3], [4] leading to a chain of human-to-human transmission in the affected area [5]. Originally only low seroprevalence rates to Ebola Zaire in humans have been observed [6], [7], [8], but recent data using a large cohort and a more sensitive approach showed that seroprevalence in central Africa might be higher than previously assumed [9]. In addition, multiple studies have shown that fruit bats, a believed source of filovirus transmission, also have seroprevalence rates from 1–3% for both Ebola and Marburg viruses [3], [10], [11], [12]. These data and the observation that lethal outbreaks have occurred more frequently in recent years [13] illustrate the urgent need for a vaccine to provide protection against all major filovirus species.

Current promising vaccine candidates against Ebola or Marburg viruses are based on adenovirus (Ad) 5 [14], [15], [16], [17], human parainfluenza virus (hPIV) type 3, venezuelan equine encephalitis virus, vesicular stomatitis virus (VSV) and virus-like particles (VLP), as vector systems that express filovirus glycoprotein, nucleoprotein, and/or VP40. All of these vaccine candidates can provide protection against otherwise lethal hemorrhagic fever in experimental infection of NHP with Marburg or Ebola virus, or both (for reviews, see [18], [19], [20]). However, several studies using different vaccine platforms have shown, that the filovirus glycoprotein is sufficient to provide protective immunity [14], [15], [16], [21], [22], [23].

Although these vaccine candidates have elicited protective immunity in NHP, their use as vaccine in humans may be limited. VSV and hPIV-type 3 are both replication competent vaccines and therefore pose a potential safety concern [18]. A VEEV based vaccine candidate expressing the filovirus nucleoprotein and/or glycoprotein did not protect against Ebola Zaire infection, but only against Marburg Musoke challenge. Albeit protective against Ebola Zaire and Marburg virus, the hurdle for VLP based vaccines is the long immunization schedule in combination with the need for adjuvants, and likely the large scale production in eukaryotic cells that is required for clinical use. Although recombinant (r)Ad5 based vaccines do not suffer from these drawbacks, as they are safe in use, can be produced in large quantities, and protect against multiple Marburg and Ebola strains, the high seroprevalence at relatively high titers due to natural infection with Ad5 [24], [25], [26] limits the use of rAd5 as a vaccine in humans. Adenoviral vectors that are based on the serotypes Ad35 and Ad26 that have a much lower seroprevalence [24], [25], [26] than Ad5. Depending on origin of subjects studies seroprevalence for Ad26 ranges from less than 10% in Northern America to 68% in Africa and for Ad35 from less than 5% in Northern America to 17% in Africa being significantly lower than for Ad5 (38% in Northern America and up to 90% in Africa). Ad26 and Ad35 seropositive subjects have furthermore lower virus neutralizing antibody titers than Ad5 which would otherwise hamper the use as vaccine vectors [27]. Ad26 and Ad35 are not affected by Ad5 pre-existing immunity [28], have been shown to induce effective humoral and cellular immunogenicity against several infectious diseases [29], [30], [31], [32], and have shown a good safety profile in humans at doses up to 10^11^ vp. More recently we demonstrated protection after immunization in a heterologous Ad26–Ad35 prime-boost regimen in NHP against homologous challenge with Ebola Zaire [33].

Most filovirus vaccine candidate efficacy studies in NHP evaluate protection, but rarely demonstrate a correlative immune response. So far the only correlate of protection against Ebola Zaire infection established in NHP studies are serum IgG levels [34]. Cellular immune responses are usually low and have only been shown to correlate with protection in one vaccine study in which depletion of CD8 lymphocyte abrogated the vaccine effect [35]. Antibodies alone will likely not provide protection as passive antibody transfer studies in NHP using either monoclonal antibody, serum from survivors, or equine hyperimmune serum failed to protect so far [36], [37], [38], [39]. In contrast, a report describing homologous passive polyclonal antibody transfer in rhesus macaques showed full protection in Marburg and Ebola virus NHP challenge studies [40]. A successful Ebola vaccine therefore likely needs to elicit both efficient humoral and cellular anti-filovirus immunity and a cellular component would also be beneficial for a Marburg vaccine.

In our present study we aimed to first assess and fully characterize the immune responses elicited by Ad35 and Ad26 vectors as vaccine platform against multiple filoviruses. To this end we constructed rAd26 and rAd35 vectors expressing the glycoproteins of five selected filovirus strains (Ebola Zaire, Ebola Sudan/Gulu, Ebola Ivory Coast, Marburg Angola, or Marburg Ravn) and evaluated these vectors for their ability to induce strain specific and cross-reactive cellular and humoral immune responses in a mouse model.

## Methods

### Ethics statement

All animal work was performed according to the Dutch law (Dutch Animal Experimentation Act) and Guidelines on the Protection of Experimental Animals by the Council of the European Committee (EU Dir. 86/609) after approval by the Dier Experimenten Commissie (DEC) of Crucell under permit numbers CRH0148 and CRH0159.

### Phylogenetic analysis

The protein sequence of the filovirus glycoprotein was derived from the GenBank database (Accession numbers: ACI28624, Q66810, Q1PD50, ABE27085, ABE27078, ABE27092, P35254, Q6UY66, P35253, Q1PDC7, Q7T9D9, Q66814, Q66798, Q05320, P87671, O11457, P87666, AAL25818). Nucleotide sequences (AY526098, AY526099, AY526100, AY526101, AY526102, AY526103, AY526104, and AY526105) of the Ebola glycoproteins collected during an Ebola Zaire outbreak from 2001–2003 in Congo and Gabon [4] were adjusted for an additional adenosine that is normally introduced by the viral polymerase to allow expression of full length glycoprotein, and translated into the corresponding protein sequence. Glycoprotein amino acid sequences were aligned with the Muscle [41] algorithm and the CLC workbench 5.7 suite was used to generate the phylogenetic tree. The reliability of the phylogenetic tree was confirmed with a bootstrap analysis using 1000 replicates with the unweighted pair group method with arithmetic mean algorithm. The tree represented is the one with the highest likelihood score.

### Adenoviral vector construction

Replication-incompetent, E1/E3-deleted recombinant adenoviral vectors based on adenovirus type 26 and 35 were engineered using the AdVac® system. Rescue and manufacturing of the replication deficient adenoviral vectors were performed in the complementing cell line PER.C6® [24], [42].

The transgene sequences encoding the different glycoproteins (GP) of the Ebola and Marburg Filoviridae, were inserted in the E1-position of the Ad genome and the coding sequences were optimized for efficient expression in mammalian cells and were placed under transcriptional control of the human CMV promoter and the SV-40 polyadenylation sequence. This expression cassette was cloned into an E1-deleted-Adapter plasmid containing the left portion of the adenoviral genome including the left inverted terminal repeat (ITR) and packaging signal. Co-transfection of this Adapter plasmid with a cosmid containing the remainder of the Ad26/35 adenoviral sequence (lacking the E3-gene and including the Ad5 E4orf6) yielded the recombinant, E1/E3-deleted, replication-deficient vaccine vectors.

Once transfected, a single plaque for each of the rAd EBOV vectors was purified and expanded up to a production scale. A two step cesium chloride gradient ultracentrifugation procedure was used to purify the rAd vectors, which were stored as single use aliquots below −65°C. Virus particle titers were quantified by measurement of optical density at 260 nm [43] and infectivity was assessed by TCID50 on the human helper cell line 911 [44]. Adenovirus-mediated GP expression was assessed by infection of A549 cells followed by analysis of culture lysates on western blot. These A549 cells were chosen as these cells lack E1A expression and therefore do not allow interfering adenoviral replication. These cells have been utilized in the past for evaluation of multiple Ad26 and Ad35 vectors where in vitro expression correlated with in vivo immunogenicity. The identity of the purified vectors was confirmed through PCR and the complete transgene sequence, including flanking regions, was verified by DNA sequencing. rAd26.empty and rAd35.empty vectors were produced similarly, but do not contain a transgene in the E1 region.

### Mice and immunizations

Six- to eight-weeks-old specific pathogen-free female BALB/c (H-2D) mice were purchased from Harlan (Zeist, The Netherlands) and kept at the institutional animal facility under specified pathogen-free conditions during the experiments. For the immunological studies, mice were vaccinated intramuscularly (i.m.) in the quadriceps of both hind legs (50 µl/leg) with the indicated vector particle (vp) dose. 10^10^ vp was used as highest dose in this animal model to avoid potential toxicity that may also interference with the immune response. Serum was obtained by heart puncture under isoflorane anesthesia and spleens were removed aseptically after cervical dislocation.

### Glycoprotein production

For production of the GP spike protein, the transmembrane domain and flanking cytoplasmic tail of the GP was deleted to ensure secretion of the glycoproteins into the supernatant and eliminate membrane insertion. The GP-ΔTm sequences were cloned into the pcDNA3.1 expression plasmids (Invitrogen) and subsequently used for transient transfection of human embryonic kidney 293T (Hek293T) cells in either T175 or T175III format. Culture supernatant was harvested and replenished with fresh medium at 4 to 8 days post transfection to obtain the GP protein, which was stored below −65°C. The glycoprotein yield was analyzed by western blot analysis.

The humanized GP sequences chosen for the adenoviral transgenes stemmed from sequences of Ebola Zaire (NP_066246), Sudan Gulu (YP_138523) and Ivory Coast (YP_003815426) strains. For the Marburg transgenes the Ravn (ACD13005) and Angola (ADM72984) strain sequences were chosen.

### Humoral immune response

Filovirus-specific humoral response was determined by a modified enzyme-linked immunosorbent assay (ELISA) to a previously described one [14]. MaxisorpTM 96-well plates (Nunc-Immuno) were coated over night at 4°C with *Galanthus Nivalis* Lectin (GNA, SIGMA Aldrich) diluted in phosphate buffered saline (PBS, GIBCO) to a concentration of 10 µg/ml. Remaining lectin solution was removed and 200 µl PBS/10% Fetal bovine serum (FBS) was added for blocking at RT for 2 hours. The plates were washed 2 times with PBS/0.2% Tween20 (PBS-T). Plates were either coated with an Ebola or a Marburg strain specific GP supernatant for 1 hour at RT and then washed 6 times with PBS-T. Mouse serum samples were diluted in sample buffer 1∶25 (PBS/0.2% Tween/1% FBS) and then in a 2-fold dilution series, added to the plates and incubated at RT for 1 hour. Plates were washed 6 times with PBS-T. Bound IgG was detected with goat-anti-mouse IgG conjugated to HRP (Cayman), diluted 1∶2000 in sample buffer and incubated for 1 hour at RT. Plates were washed 6 times with PBS-T. OPD (Sigma Aldrich) was added and incubated in the dark fo 10 minutes. The reaction was stopped and measured at 492 nm. Relative serum titers were calculated against a filovirus glycoprotein strain specific reference serum.

### Cellular immune response

The number of filovirus glycoprotein-specific, interferon gamma-secreting T cells splenocytes of immunized mice was determined with an ELISPOT assay as previously described [45]. The peptide pools used for stimulation for each Ebola and Marburg strain glycoprotein consist of 15-mers overlapping by 11 amino acids. To minimize undesired effects of a too high number of peptides in a pool, each glycoprotein peptide pool was divided into two, one N-terminal and one C-terminal half. Peptides that overlap with more than nine consecutive amino acids within the three Ebola or two Marburg strains were combined in a consensus pool. The data presented shows the overall response to all reactive peptide pools. For the epitope mapping single peptides of the relevant pools were used for stimulation in the IFN-γ ELISPOT assay. The peptide pools and single peptides were used at a final concentration of 1 µg/ml for each single peptide.

### Statistical analysis

Data are presented as (geo) means. Statistical analyses were performed with SPSS version 17.0 (SPSS Software Inc., 2008). Immune responses (logarithmically transformed) among groups of animals were assessed with ANOVA and post-hoc Tukey test. For the cross reactivity of the humoral immune response an ANOVA and Dunnett post-hoc was used. Differences were considered significant when p≤0.05.

## Results

### Vaccine vector design

Filovirus outbreaks are unpredictable in nature and the potential use of filoviruses in a bioterrorism act stresses the need for a vaccine that can provide protection against all major strains. In order to obtain optimal coverage by a filovirus glycoprotein based vaccine we performed a phylogenetic analysis of all available glycoprotein amino acid sequence of all strains that caused outbreaks of hemorrhagic fever since the identification of filovirus as the causative agent in 1967 (**Figure S1**). Ebola can be subdivided into Ebola Zaire, Sudan, Ivory Coast, and Bundibugyo species whereas Marburg virus glycoprotein is more homologous in sequence and cannot be grouped in distinct virus species. The phylogenetic analysis of the glycoprotein resembles the filovirus taxonomy based on full-length nucleotide sequence diversity [46].

Accordingly, challenge studies with filovirus vaccines in NHP have shown so far that Marburg virus vaccines based on glycoprotein sequences are generally cross-protective [21], [47], whereas Ebola Zaire vaccine does not provide protection from Ebola Sudan challenge [23], although it does provide protection against the newly emerged Ebola Bundibugyo strains [15]. Thus, a vaccine to protect against all filovirus strains should at least contain the Marburg Angola, Ebola Zaire, and Ebola Sudan glycoprotein sequences. In addition, we have added Ebola Ivory Coast and Marburg Ravn glycoprotein expressing vaccine vectors to increase the chances of protection against potentially newly emerging and existing strains against which a vaccine based on Ebola Zaire, Ebola Sudan, and Marburg Angola glycoprotein may not cross-protect.

We then generated and quality controlled the rAd26 and rAd35 adenoviral vectors expressing the selected glycoproteins (**Table S1**). To control for transgene expression and processing of the inserted glycoprotein, rAd26 and rAd35 were used to transduce the susceptible human lung carcinoma epithelial cell line A549 at different doses as a model for in vivo expression. A western blot analysis showed a dose dependent expression of glycoprotein for all filovirus proteins expressed by rAd26 and rAd35 vectors (**Figure S2**).

### Humoral immune response of Balb/c mice to filovirus glycoprotein expressed in rAd26 and rAd35

**Figure 1:**
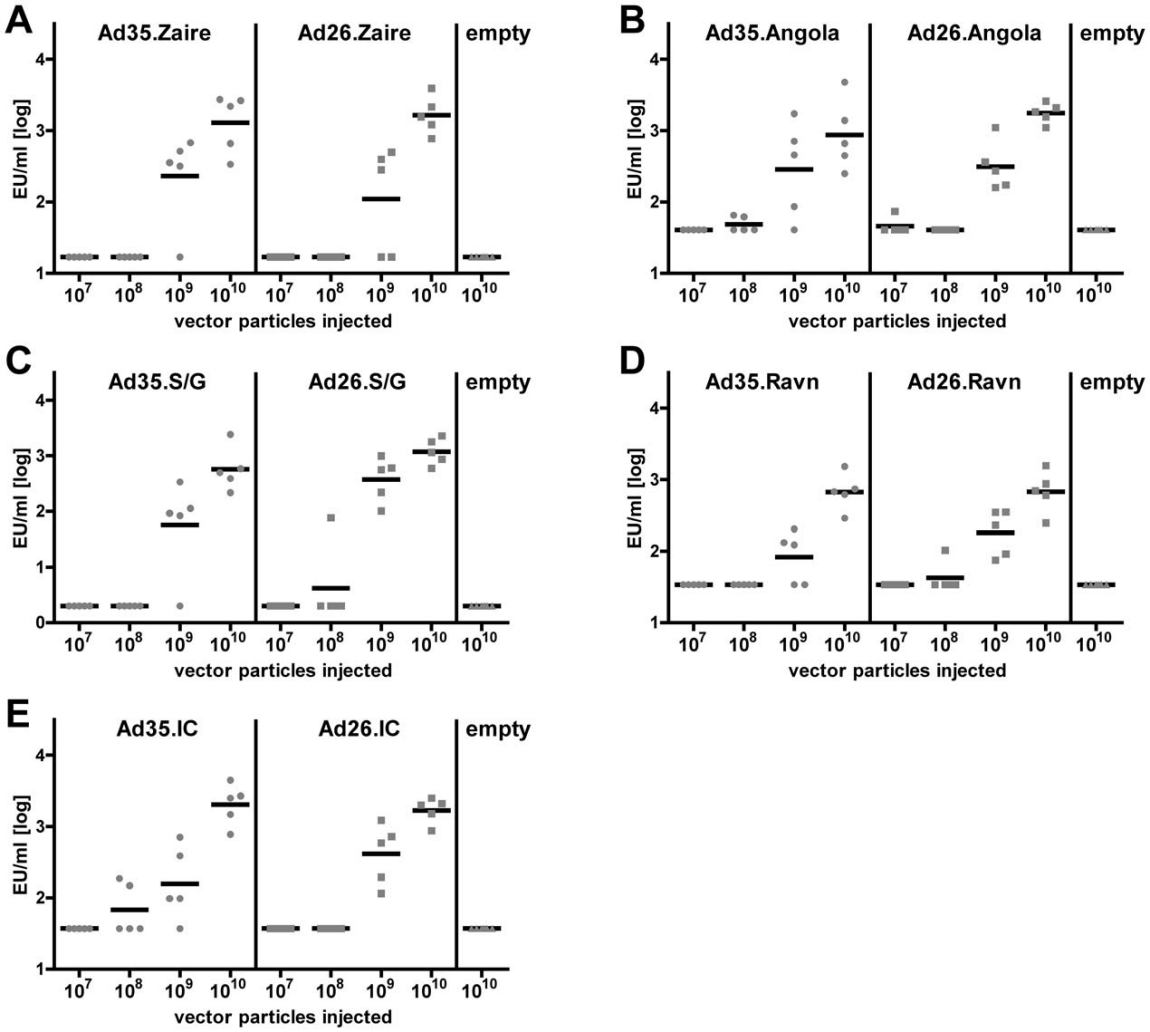
Dose dependent humoral immune response of filovirus rAd26 and rAd35 vectors. The filovirus specific humoral immune response was detected by ELISA from serum isolated from mice either vaccinated with 10^7^ to 10^10^ vp of rAd26 or rAd35 coding for Ebola Zaire (A), Ebola Sudan Gulu (C), Ebola Ivory Coast (E), Marburg Angola (B), or Marburg Ravn (D) glycoprotein (n = 5 per dose, filovirus strain and serotype). For each individual experiment three mice each were immunized with rAd26 or rAd35 without a transgene in the E1 region. Arbitrary ELISA units/ml (EU/ml) of serum samples were determined against a reference. The bar represents the mean of the log transformed EU/ml and the grey balls an individual mouse.

Serum IgG titers against filovirus glycoprotein after vaccination are the best correlate of protection against filovirus in NHP identified thus far [34], as no level of protective cellular responses have been defined yet. To assess the induction of serum IgG titers by our vaccine candidates, Balb/c mice were immunized intramuscularly with an escalating dose (10 fold increase per step, range 10^7^ to 10^10^ vector particles (vp)) of each of the rAd26 and rAd35 vectors with the glycoprotein of Ebola Zaire, Ebola Sudan/Gulu, Ebola Ivory coast, Marburg Angola, or Marburg Ravn as a transgene. The humoral immune response against the homologous antigen was measured four weeks after immunization using a mouse IgG specific sandwich ELISA that was developed for each filovirus strain glycoprotein. At a dose of 10^9^ and 10^10^ vp, a dose dependent glycoprotein specific humoral immune response was elicited by all different vectors (**Figure 1**) while, in general, no antibodies could be detected when mice were immunized with 10^7^ or 10^8^ vp. There was no significant difference in magnitude of immune responses against the different transgenes expressed by either rAd26 or rAd35 although a trend towards superiority of immune responses against rAd26 expressed genes was observed (*P* = 0.06, ANOVA).

**Figure 2:**
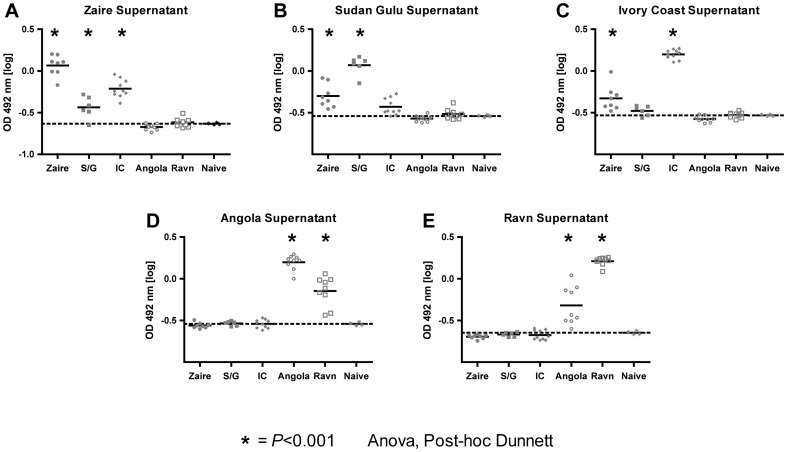
Cross reactivity of the elicited humoral immune response. The humoral immune response was detected by ELISA in serum isolated from mice either vaccinated with 10^10^ vp of rAd26 or rAd35 coding for the glycoprotein of one of the five filovirus strains. Serum from 10 mice vaccinated against one of five filovirus strain was probed against ELISA plates coated with either Ebola Zaire (A), Ebola Sudan Gulu (B), Ebola Ivory Coast (C), Marburg Angola (D), or Marburg Ravn (E) glycoprotein-containing supernatant. Naïve serum from five mice each was used as control and the average of these is represented as a dotted line. The bar represents the mean of the log transformed OD and the grey balls an individual mouse each. The star indicates a significant difference from the negative control (p<0,001, Anova, Post-hoc Dunnett).

We next investigated whether the immune response elicited by the single filovirus antigens provided cross reactive capacity. Serum from each of the ten mice that were immunized with 10^10^ vp rAd26 or rAd35 expressing the different filovirus glycoproteins under study were tested for reactivity against each of the five filovirus glycoproteins in an ELISA (**Figure 2**). As expected, antibodies elicited by the vaccine candidates expressing Ebola glycoprotein did not cross-react with Marburg glycoproteins while antibodies elicited by Marburg glycoprotein did not cross-react with Ebola glycoprotein. However, sera from mice immunized with Ebola Zaire glycoprotein expressing vectors were cross reactive with Ebola Sudan/Gulu and Ivory Coast (p<0.001, ANOVA; **Figure 2 B and C**). In contrast, sera from mice that were immunized with Ebola Ivory Coast glycoprotein expressing vectors were cross reactive with glycoprotein from Ebola Zaire (p<0.001, ANOVA) but only minimally with glycoprotein from Ebola Sudan/Gulu (**Figure 2 A and B**). Similarly, sera from mice immunized with Ebola Sudan/Gulu glycoprotein expressing vectors only cross reacted with glycoprotein of Ebola Zaire (p<0.001, ANOVA) but barely with Ebola Ivory Coast (**Figure 2 A and C**). Sera from mice that were immunized with rAd vectors expressing one of the two different Marburg strain glycoproteins were cross-reactive with the other Marburg strain (p<0.001, ANOVA; **Figure 2 D and E**). These results may suggest that an Ebola or Marburg virus glycoprotein vaccine will provide a certain degree of cross protection against viruses from the same serotype, although no general correlates of protection for filovirus vaccines have been defined up to date.

### Cellular immune response to rAd26 or rAd35 expressed filovirus glycoprotein

We next measured cellular immune responses in mice four weeks after vaccination with either rAd26 or rAd35 vectors expressing filovirus glycoprotein using an IFN-γ ELISPOT. Splenocytes of mice were stimulated for 16 hours with homologous glycoprotein 15mer peptide pools spanning the entire glycoprotein. The peptide pools were subdivided into a glycoprotein specific N-terminal pool 1 and a C-terminal pool 2. In addition, an Ebola and Marburg consensus peptide pool was constructed out of all peptides within the Ebola or Marburg strains that have an overlap of more than nine amino acids. These peptides were not part of the single filovirus glycoprotein strains pool 1 or pool 2.

**Figure 3:**
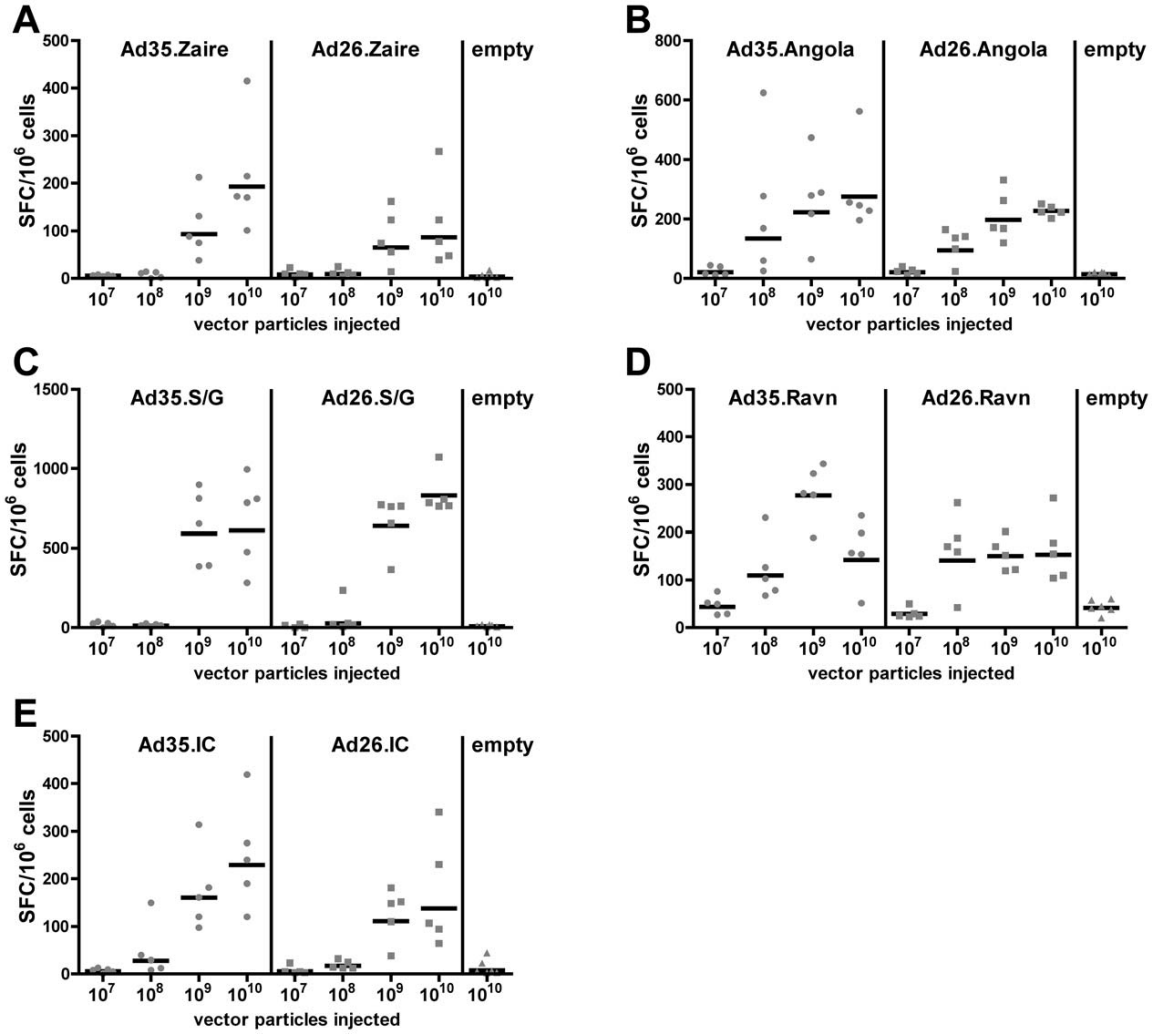
Dose dependent cellular immune response of filovirus rAd26 and rAd35 vectors. The cellular immune response was detected by ELISPOT of isolated splenocytes from mice either infected with 10^7^ to 10^10^ vp of rAd26 or rAd35 coding for the glycoprotein Ebola Zaire (A), Ebola Sudan Gulu (C), Ebola Ivory Coast (E), Marburg Angola (C), or Marburg Ravn (D). For each individual experiment three mice each were immunized with rAd26 or rAd35 without a transgene in the E1 region. Displayed is the total number of strain specific filovirus glycoprotein responsive splenocytes in spot forming colonies per million cells (SFC/10^6^ cells). The bar represents the geometric mean of five mice and the grey balls an individual mouse.

Similar to the humoral immune response, mice immunized with one of the three Ebola glycoproteins elicited a cellular immune response in a dose dependent manner with the highest response elicited by 10^10^ vp, a lower response at 10^9^ vp and no or very minor response at 10^8^ vp or 10^7^ vp (**Figure 3 A, C, E**). Interestingly, the Ebola Sudan/Gulu glycoprotein expressing vector elicited a more than 3-fold higher response than the Ebola Zaire and Ivory Coast glycoproteins. There was no effect of the adenoviral vector serotype on the magnitude of the response in the highly responsive Ebola Sudan/Gulu mice (p = 0.249, ANOVA) (**Figure 3 C**). However, cellular immune responses against Ebola Zaire and Ivory Coast glycoproteins tended to be slightly higher when expressed in rAd35 than when expressed in rAd26 (Ebola Zaire: p = 0.092; Ebola Ivory Cost: p = 0.110; ANOVA) (**Figure 3 A and E**). Cellular immune responses were only detected after stimulation with peptide pool 1 (data shown in **figures 3 A, C, E**), whereas no reactivity against peptide pool 2 and the Ebola consensus pool was observed in mice immunized with any of the Ebola glycoproteins (data not shown).

In contrast to the Ebola vectors, both Marburg vectors elicited a cellular immune response already at a dose of 10^8^ vp (**Figure 3 B and D**). The T cell response in **figure 3 B and D** shows the combined reactivity against peptide pool 2 and the Marburg consensus pool. For peptide pool 1 no reactivity was found (data not shown). The majorities of T cells were reactive against the consensus pool and to a lesser extend against peptide pool 2, which indicates a high degree of cellular cross reactivity between the Marburg strains. For Marburg Angola a dose-dependent response from 10^7^ to 10^10^ vp was observed. In the case of Marburg Ravn all vector doses from 10^8^ to 10^10^ vp elicited a cellular immune response, but no clear dose response was observed (**Figure 3D**). Overall the strength of the response of both Marburg antigens was similar to the Ebola Zaire and Ivory Coast antigens, with similar T cell responses for rAd35 than rAd26 (Marburg Angola: p = 0.390, Marburg Ravn: p = 0.382, ANOVA).

### Filovirus glycoprotein T cell epitope characterization

To better characterize and understand the cellular immune response elicited in BALB/c mice by the rAd26 and rAd35 filovirus vaccine candidates we used a two tiered strategy. In a first immunization experiment we screened vaccinated mice, using an IFN-γ ELISPOT assay, for reactivity against peptide pools that consisted of a mixture of single 15-mer peptides overlapping by eleven amino acids (data not shown). In a second immunization experiment, splenocytes from vaccinated mice were stimulated in the IFN-γ ELISPOT with single peptides from the reactive peptide pools. The response against a peptide was considered positive when it was higher than the average plus three times standard deviation of the response against medium alone. For each filovirus strain we identified specific peptides that contain a T cell epitope (**Table 1**). For Ebola Zaire we identified one peptide containing a dominant T cell epitope that was identified previously [48]. In the case of Ebola Ivory Coast two peptides were identified that presented similar (66% amino acid identity), but not identical, sequences as the peptide identified for Ebola Zaire, with matching H-2L^D^ anchoring amino acids (G**P**CPGGLA**F**HK). Similar to the high reactivity in the ELISPOT with peptide pool 1, Ebola Sudan/Gulu reactive splenocytes were also highly reactive against two peptides that allowed mapping of the dominant epitope to the amino acid sequence DR**P**HTPQFL**F**Q of an H-2L^D^ MHC1. Interestingly, a similar sequence in potential 9-mer MHC1 binding sequence or MHC1 anchoring motifes did not necessarily elicit a similar reactivity within the three Ebola strains (**Table 1**).

**Table 1:** Cross reactivity of identified murine T cell epitopes.

Strain	Peptide Sequence	Peptide ID	Reactivity	IC50
Zaire	VSGTG**P**CAGDFA**F**HK	P28	High	H-2L_D_ 12 nM
	G**P**CAGDFA**F**HKEGAF		No	H-2L_D_ 12 nM
Ivory Coast	VSGTG**P**CPGGLA**F**HK	P28	High	H-2L_D_ 489 nM
	G**P**CPGGLA**F**HKEGAF	P29	High	H-2L_D_ 489 nM
Sudan Gulu	AQGTG**P**CPGDYA**F**HK		No	H-2L_D_ 155 nM
	G**P**CPGDYA**F**HKDGAF		No	H-2L_D_ 155 nM
Zaire	DNLTYVQLESRFTPQ		No	Above 5000 nM
	YVQLESRFTPQFL**L**Q		No	H-2L_D_ 2502 nM
	ESRFTPQFL**L**QLNET		No	H-2L_D_ 2502 nM
Ivory Coast	DHLTYVQLEARFTPQ	p52	Low	Above 5000 nM
	YVQLEARFTPQFLVL		No	Above 5000 nM
	EARFTPQFLVLLNET		No	Above 5000 nM
Sudan Gulu	DNNTFVRLDRPHTPQ		No	Above 5000 nM
	FVRLDR**P**HTPQ**F**LFQ	P53	High	H-2L_D_ 13 nM
	DR**P**HTPQFL**F**QLNDT	P54	High	H-2L_D_ 13 nM
Zaire	VIYRGTTFAEGVVAF		No	Above 5000 nM
	GTTFAEGVVAFLILP	p36	Low	Above 5000 nM
Ivory Coast	IIYRGTTFAEGVIAF	p35	Low	Above 5000 nM
	GTTFAEGVIAFLILP		No	Above 5000 nM
Sudan Gulu	VIYRGVNFAEGVIAF		No	Above 5000 nM
	GVNFAEGVIAFLILA	p36	Low	Above 5000 nM
Zaire	L**Y**DRLASTVIYRGTT	p33	Low	H-2K_D_ 54 nM
Ivory Coast	L**Y**DRLAST**I**IYRGTT		No	H-2K_D_ 54 nM
Sudan Gulu	L**Y**DRLASTVIYRGVN		No	H-2K_D_ 08 nM
Zaire	TKKNLTRKIRSEELS	p66	Yes	Above 5000 nM
Ivory Coast	NKKNFTKTLSSEELS		No	Above 5000 nM
Sudan Gulu	NKKNLSEQLRGEELS		No	Above 5000 nM
Angola	GGTCKVL**GP**DCCIG**I**	p146	High	H-2L_D_ 69 nM
Ravn	GGTCKVL**GP**DCCIG**I**	p146	High	H-2L_D_ 69 nM
Angola	VFTEGNIAAMIVNKT		No	Above 5000 nM
Ravn	VFTEGNIAAMIVNKT	p36	Low	Above 5000 nM
Angola	*HLV***Y**FR*R*KR*N***I***L*W*R*E	p103	Low	H-2K_D_ 110 nM
Ravn	*PPI***Y**FR*K*KR*S***I***F*W*K*E	p103	No	H-2K_D_ 57 nM

The Sequence of reactive peptides identified is given and peptides with homologous sequences within the Ebola and Marburg strains are displayed for comparison. Underlined sequences are the predicted [65] MHC class 1 9mer binding sequence using the ANN algorithm [65], the binding strength to the specific MHC class 1 is given in the last column and was calculated using the T cell epitope prediction tool of the IEDB database (http://www.immuneepitope.org/). Amino acids in bold label the anchoring amino acids of the 9mer according to Rammensee et al. [66].

For both Marburg strains, one peptide that is identical in sequence between the Marburg Angola and Ravn strains was mapped to contain a T cell epitope. Another peptide, p103, shares 77.7% amino acid identity of the 9-mer MHC1 binding motife between the Marburg Angola and Ravn, however only Marburg Angola was responsive to this peptide. An additional reactive peptide was identified for Marburg Ravn, but not for Marburg Angola. These results were confirmed for all peptides in a separate experiment (data not shown). For none of the filovirus antigens a significant difference between rAd26 and rAd35 to induce peptide reactive T cells was observed other than in the strength of the response, similar to the peptide pool reactivity.

### Humoral and cellular immune response to heterologous prime-boost

Several studies have shown that rAd26 and rAd35 as components of a heterologous prime-boost regimen elicit a sustained T cell and humoral immune response against various antigens [29], [30], [31], [32]. We have shown so far, that immunization with a single dose of 10^10^ vp rAd26 and rAd35 elicits a potent humoral and cellular immune response against filovirus glycoproteins in mice. Encouraged by these results we evaluated if a heterologous prime-boost using rAd26 and rAd35 could further improve the filovirus glycoprotein specific humoral and cellular immune responses. We did not test a homologous prime-boost, since we have shown before for adenoviral vectors that compared to a heterologous regimen such a vaccination regimen only modestly increases the immune response in mice and NHP due to an anti-vector immune response [31], [32], [49]. As prototype filovirus strains we used Ebola Zaire and Marburg Angola coding vectors in two separate experiments. Balb/c mice were immunized with one dose of 10^10^ vp of rAd26.Ebo(Z), a rAd26 coding for Ebola Zaire glycoprotein, and four weeks later boosted with 10^10^ vp of heterologous rAd35.Ebo(Z). Another group was immunized first with rAd35.Ebo(Z) and four weeks later with rAd26.Ebo(Z) at the same dose. As a single vector immunization control, mice were immunized with rAd26.Ebo(Z) or rAd35.Ebo(Z) and boosted with rAd35.empty or rAd26.empty, respectively. Negative control groups received either rAd26.empty in combination with a boost of rAd35.empty or vice versa. For Marburg Angola a similar experimental strategy was used. The vaccine induced response was evaluated either at two or eight weeks post vaccination, to capture the acute and memory phase of the induced humoral and cellular immune response.

**Figure 4:**
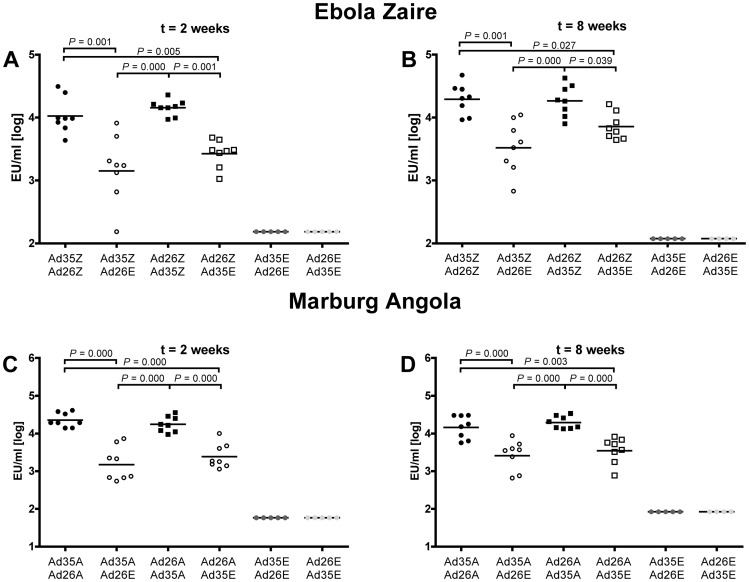
Enhanced filovirus specific humoral immune response to a rAd26/rAd35 prime-boost regimen. The filovirus specific humoral immune response was detected by ELISA. Serum was isolated from groups of mice at two or eight weeks post boost vaccination with 10^10^ vp of with vectors coding for Ebola Zaire (A and B), Marburg Angola (C and D), or as control for prime only with a no-antigen coding control vector. The black filled circles represent the group of Ad35.Ebo(Z) or Ad35.Mar(A) primed and Ad26.Ebo(Z) or Ad26.Mar(A) boosted animals; black open circles represent the Ad35.Ebo(Z) or Ad35.Mar(A) primed animals which received the no-antigen coding control vector Ad26.empty as boost. The black filled squares represent the group of Ad26.Ebo(Z) or Ad26.Mar(A) primed and Ad35.Ebo(Z) or Ad35.Mar(A) boosted animals; black open squares represent the Ad26.Ebo(Z) or Ad26.Mar(A) primed animals which received the no-antigen coding control vector Ad35.empty as boost. The dark grey circles represent the control animal group receiving Ad35.empty for prime and Ad26.empty as boost and the light grey circles the animal group receiving Ad26.empty for prime and Ad35.empty as boost. The bar denotes the mean of the log transformed sample titers of eight mice (five for the control groups). The p values given are calculated by Anova and a Post-hoc Tukey test.

The humoral immune response elicited by the prime-boost regimen was significantly higher than the immune response elicited by single filovirus antigen expressing rAd26 or rAd35 (p<0.039, ANOVA; **Figure 4**). One log higher humoral immune responses were observed at both two and eight weeks post immunization for both Ebola Zaire and Marburg Angola. We did not detect a difference between the heterologous prime-boost regimens with either rAd26 prime and rAd35 boost or rAd35 prime and rAd26 boost. Importantly, we did find a sustained high humoral immune response for both Ebola Zaire and Marburg Angola study arms at eight weeks post boost. We did not observe a loss in antibody titer between week two and eight after the boost. Interestingly, the variation in antibody titer within the prime-boost groups was lower than within the single immunization group. This lower variation in the induced humoral immune response indicates that the heterologous prime-boost regimen induces a more homogenous humoral immune response, independent of potential differences between animals.

**Figure 5:**
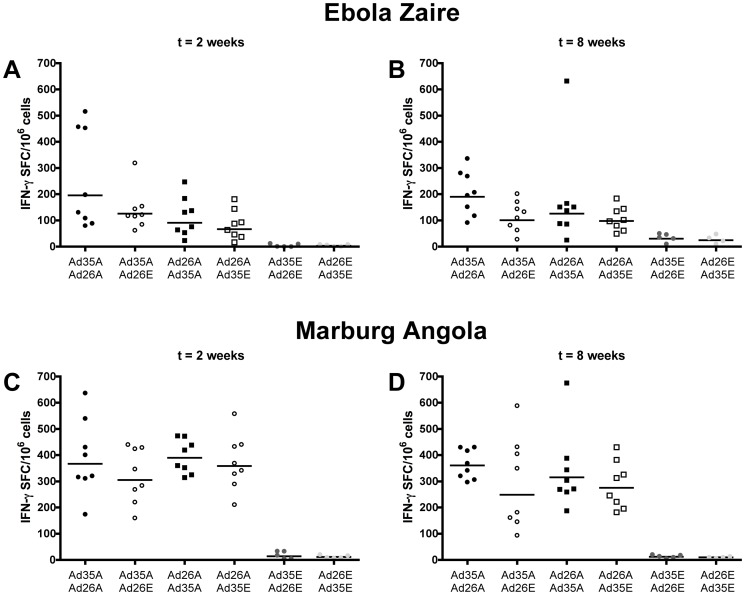
Enhanced filovirus specific cellular immune response of a rAd26/rAd35 prime-boost regimen. Filovirus specific cellular immune response was detected by ELISPOT after stimulation with peptide pools spanning the entire glycoprotein. Splenocytes were isolated from groups of mice at two or eight weeks post boost vaccination with 10^10^ vp of with vectors coding for Ebola Zaire (A and B), Marburg Angola (C and D), or as control for prime only with a no-antigen coding control vector. The black filled circles represent the group of Ad35.Ebo(Z) or Ad35.Mar(A) primed and Ad26.Ebo(Z) or Ad26.Mar(A) boosted animals; black open circles represent the Ad35.Ebo(Z) or Ad35.Mar(A) primed animals which received the no-antigen coding control vector Ad26.empty as boost. The black filled squares represent the group of Ad26.Ebo(Z) or Ad26.Mar(A) primed and Ad35.Ebo(Z) or Ad35.Mar(A) boosted animals; black open squares represent the Ad26.Ebo(Z) or Ad26.Mar(A) primed animals which received the no-antigen coding control vector Ad35.empty as boost. The dark grey circles represent the control animal group receiving Ad35.empty for prime and Ad26.empty as boost and the light grey circles the animal group receiving Ad26.empty for prime and Ad35.empty as boost. The bar denote the geometric mean of the responsive cells of eight mice (five for the control groups).

The cellular immune response to the heterologous prime-boost was analyzed by IFN-γ Elispot. The prime-boost regimen for Ebola Zaire showed a pattern of higher immune responses than the respective single immunization at two and eight weeks post boost whithout reaching statistical significance or a clear superiority of Ad26 or Ad35 as priming vector (**Figure 5 A and B**). Analyzing the time points combined we found an increased reactivity of the prime-boost regimen than the respective single immunization (combined time points: p = 0.017; ANOVA). The reactive peptide pools found in the prime-boost are similar to the ones found with either single immunization of rAd26.Ebo(Z) or rAd35.Ebo(Z) and a response against peptide 28 was detected (data not shown) that mimics the response to peptide pool 1 on a lower level.

rAd26 and rAd35 expressing Marburg Angola glycoprotein were also not different in eliciting a cellular immune response if used as priming vector in the heterologous prime-boost regimen (**Figure 5 C and D**). For Marburg Angola, the pattern in the immune response elicited by prime-boost directly compared to the respective single immunization was more obvious, but again significantly superior only if analyzed combined across time points (combined time points: p = 0.037; ANOVA). In figure **5** the combined reactivity against pool 2 and the consensus pool is shown. Again, a higher reactivity against the consensus peptide pool than peptide pool 2 was detected. The single peptide p103 in pool 2 and p146 in the consensus pool both showed a similar reactivity as what was observed after single prime immunization.

The cellular immune response is not boosted to a similar extend as the humoral immune response with this heterologous prime-boost regimen for both, Ebola Zaire and Marburg Angola. However, similar to the humoral immune response, the adenoviral vaccination regimens induced a cellular immune response that remained stable between weeks two and eight post boost. For the Ebola Zaire vaccine candidate this was observed for all vaccination regimens. However, for Marburg Angola the cellular immune response seemed to be more stable when elicited by the prime-boost regimens compared to mice that only received a prime.

## Discussion

Filovirus hemorrhagic fever outbreaks are sporadic and can be caused by diverse known or newly emerging Ebola and Marburg strains. A vaccine inducing a protective immune response against all known highly lethal strains is of utmost importance for the protection of the population in affected areas, researchers, health care workers and other personnel employed to the outbreak area. After phylogenetic analysis of Ebola sequences, we have chosen the glycoprotein genes of three Ebola viruses and two Marburg virus strains to be included in our vaccine, in an attempt to achieve highest breadth of protection. These five filovirus glycoproteins in the background of our rAd26 and rAd35 vectors were highly immunogenic in mice, eliciting both humoral and cellular immune responses.

Our phylogenetic analysis showed that in the decades between the different repeated outbreaks of Ebola Zaire, Ebola Sudan, and Marburg virus none of the viruses had accumulated high amino acid sequence diversity in the glycoprotein. The genetic stability of filoviruses, not only in the glycoprotein, but throughout the viral genome [50], [51], [52] is higher than expected from the high sequence diversity and mutation rate of other negative strand RNA viruses [53], [54], [55]. One of the factors contributing to the high genetic stability observed in the virus isolated from humans may be the zoonotic transmission bottleneck that only allows for transmission of certain virus quasispecies. Since it is not to be expected that new outbreaks will be caused by highly divergent filoviruses, our pentavalent vaccine may have the potential of protecting against new variants of the virus strains that are already included in our pentavalent vaccine candidate. Indeed, several studies have shown that a Marburg virus glycoprotein based vaccine can elicit cross protective immunity against two heterologous Marburg strains [21] and that a vaccine based on Ebola Zaire glycoprotein can elicit cross protective immunity against Ebola Bundibugyo [15].

This capacity of one filovirus glycoprotein to cross-protect against a more distant strain is also implicated by our results. Sera from mice vaccinated with rAd26.Ebo(Z) and rAd35.Ebo(Z) is cross-reactive with Ebola Ivory Coast and Sudan/Gulu glycoprotein, but to a lower extend than homologous serum. This is in agreement with an earlier study that has shown that Ebola Zaire glycoprotein expressed by recombinant VSV is not able to protect against Ebola Sudan challenge in a NHP filovirus challenge model [23]. Sera from rAd26.Ebo(IC) and rAd35.Ebo(IC) immunized mice were minimally cross-reactive with Ebola Zaire glycoprotein but not with Ebola Sudan/Gulu glycoprotein while sera from mice that received rAd26.Ebo(S/G) or rAd35.Ebo(S/G) had a relatively low degree of cross-reactivity against Ebola Zaire but not Ebola Ivory Coast. Altogether, these data indicate that Ebola Zaire alone potentially provides the broadest protection albeit still minimally.

Sera from mice that were immunized with rAd26.Mar(A) or rAd35.Mar(A) were cross reactive with Marburg Ravn glycoptrotein and vice versa while the cellular immune response in these mice was mainly directed against the consensus peptide pool of the Angola and Ravn strains. Based on the cross reactivity between Marburg Angola and Ravn of both the humoral and cellular immune responses it is likely that one vaccine strain will be sufficient to protect against all Marburg strains as has been shown in a NHP filovirus cross-protection study [21]. The understanding of the cross reactivity of Ebola and Marburg sera observed here will be extended in the future in NHP filovirus challenge studies to fully evaluate the degree of cross protection provided by the rAd26 and rAd35 vectors described here.

It has been reported that the rAd26 and rAd35 utilize distinct cellular receptors for viral entry [24]. Human CD46, a receptor that is used by rAd35 and to a lesser extend by rAd26, has no equivalent in mice. However, in our studies using Balb/c mice that do not express the human CD46 receptor the potency of the humoral immune response did not differ between rAd26 and rAd35. This was independent of the filovirus glycoprotein strain and the vaccination dose used. In a recent NHP Ebola Zaire vaccine study, rAd35 vaccination elicited a lower humoral immune response than rAd26 vaccination and provided a lower degree of protection from homologous challenge [33]. Importantly, we here achieved a strong anti-filovirus glycoprotein antibody response when rAd35 or rAd26 was used as a priming vector in the heterologous prime-boost in mice, for both Ebola Zaire and Marburg Angola expressing vectors. Such a boosting effect on the Ebola Zaire humoral immune response was also seen in a rAd26-prime/rAd35-boost regimen in NHP [33]. Likely, a similarly improved humoral immune response in NHP may be expected for the other Ebola and Marburg strains used in our current study. These data underline the improved immunogenicty of heterologous prime-boost vaccine regimens as has been observed for other infectious disease vaccines [30].

The degree of protection from filovirus challenge that can be provided by the cellular immune response has so far not been studied in great detail and the magnitude and quality of responses that are needed for protection are mostly unknown. Furthermore, data on the cellular immune response after outbreaks in humans is very limited [56]. A major advantage of adenoviral vaccines is that they not only induce the “classical” humoral immune response, but also cellular immune responses. This has been shown for several adenovirus-based vaccines [24], [28], [29], [31], [32], [49], [57], [58]. Passive antibody transfer studies so far have indicated that antibodies alone will very likely not be sufficient to provide protection against all filovirus infections [35], [36], [37], [38], [39], with one recent study showing a promising result in providing full protection against Marburg and Ebola challenge after transfer of polyclonal serum [40]. However, for a vaccine to provide protection the induced cellular immune response is very likely indispensable. It has been shown for one adenoviral based filovirus vaccine that depletion of CD8+ T cells abrogates full protection from disease despite maintaining a similar high humoral immune response as surviving NHP [35]. In this manuscript we have demonstrated the ability of rAd26 and rAd35 based vectors to induce a uniform high filovirus specific cellular immune response in mice for all five filovirus strains tested. The rAd vaccine vectors expressing Ebola Sudan/Gulu elicited an even higher cellular immunogenicity than those expressing the other four glycoproteins. This high reactivity is mainly induced by two overlapping peptides that contain one predicted CD8+ T cell epitope with a high binding affinity to H-2L^D^ MHC1. As the cellular immune response is biased in breadth in inbred mice we expect a higher breath of the response to Ebola Sudan/Gulu in NHP and in humans, which have a more diverse and complex MHC1 repertoire.

We failed to see a clear dose dependency for the cellular immune response which was different from the humoral immune response to insert and vector which were both dose dependent. It cannot be excluded that the strength of the cellular immune response depends on the antigen specific naïve T cell precursor frequency [59], [60]. A higher antigen exposure and presentation may not necessarily lead to a higher cellular response if the maximum expansion capacity of the cells is reached. In addition, it was recently shown that Ebola Zaire GP sterically hinders access to the MHCI of antigen presenting cells [61], [62], which may be a general mechanism that applies to all filovirus glycoproteins. This mechanism would be rate limiting for the cellular but not the humoral immune response. These effects may also influence the heterologous rAd26 and rAd35 prime-boost regimen for Marburg Angola and Ebola Zaire that did only sub-optimally improve the cellular immune response in contrast to the clearly improved humoral immune response. However, a relatively sustained cellular immune response after heterologous prime-boost and single vector immunization was observed for up to 12 weeks post initial vaccination. This indicates that the generated cellular immune response is not comprised mainly of short lived effector cells, but of memory cells that may be able in conjunction with the humoral immune response to provide long term protection from infection.

In this manuscript we have shown the high immunogenicty of a rAd26 and rAd35 based filovirus vaccine candidate for five filovirus strains and the enhanced immunogenicty of these recombinant adenoviral vectors when combined in a heterlogous prime-boost. Such a pentavalent adenoviral vector based vaccine would potentially protect against a wide range of filovirus strains. So far, the combination of glycoproteins of different filovirus strains did not interfere with optimal immune responses against each individual component, nor did it affect protection of NHP against filovirus challenge, as compared to the protection achieved with a monovalent vaccine [14], [15], [17], [23], [63], [64]. Results obtained here thus warrant for the evaluation of a combined pentavalent heterologous prime-boost vaccine in future NHP studies to provide protection against multiple filoviruses.

## Supporting Information

Table S1 **Characterization of adenoviral vectors.** All adenoviral vectors were characterized by determination of vector particle (VP) to infectious units (IU) ratio, transgene sequencing, and expression by western blot. All vectors were purified by Caesium chloride (CsCL) density purification.
(PDF)

Figure S1 **Phylogenetic relationship of the major filovirus strains that have caused outbreaks.** Phylogenetic tree of the filovirus glycoprotein of all major filovirus strains using the UPGMA method. Confidence values are displayed at internal branches as percent of 1000 bootstraps. The branch length represents the phylogenetic distance. For better visualization the Zaire and Sudan strains are underlayed in grey. The tree was constructed with the Muscle algorithm for protein sequence alignment and the CLC workbench software for drawing of the tree. Accession numbers of the sequences used are described in material and methods. The numbers behind the strain names are the last two digits of year of strain isolation. Ebola Reston was not included as so far no human cases have been reported.
(PDF)

Figure S2 **Filovirus glycoprotein expression by rAd26 and rAd35 vectors.** Western blot analysis of A549 cells cultured to 70% confluency in 24-well plates infected with either rAd26 or rAd35 coding for either Ebola Zaire, Ebola Sudan/Gulu, Ebola Ivory Coast, Marburg Angola, or Marburg Ravn (lane 1–3 at an multiplicity of infection (MOI) of 10000, 25000, or 50000 for rAd26 and at an MOI of 1000, 2500, or 5000 for rAd35 vectors). MOI were based on vp/cell and in vitro transduction efficacy of Ad26 is lower than for Ad35 for A549 cells which was adjusted for by using a 10-fold higher MOI. The positive controls are cells infected with rAd5 (MOI 5000) coding for the same antigen (lane 7). The murine serum to detect the antigens is isolated out of Balb/c mice i.m. injected with rAd5 vectors four weeks before. The in this way generated Ebola specific sera were predominantly reactive against the GP1 (or/and GP0), whereas the Marburg specific sera specifically reacted with sera against GP2 in the western blot. Negative controls are untreated (lane 6) and rAd35.empty or rAd26.empty vector (lane 5, MOI of 50000 for rAd26 and MOI of 5000 for rAd35) infected A549 cells. Lane 4 is loaded with molecular weight marker.
(PDF)
