# Supplementary material for: Ad35 and Ad26 Vaccine Vectors Induce Potent and Cross-Reactive Antibody and T-Cell Responses to Multiple Filovirus Species
Source: PLoS One. 2012 Dec 6;7(12):e44115. doi: 10.1371/journal.pone.0044115 (PMC3516506; doi:10.1371/journal.pone.0044115)
Supplement: Table S1 — Characterization of adenoviral vectors. All adenoviral vectors were characterized by determination of vector particle (VP) to infectious units (IU) ratio, transgene sequencing, and expression by western blot. All vectors were purified by Caesium chloride (CsCL) density purification. (PDF) [file pone.0044115.s001.pdf]

Supplemental table 1:

Vector Name	Lot number	VP/IU ratio	Transgene sequence*	Purification method	Transgene expression
Ad35.Ebo(Z)	G430-016	10	confirmed	CsCl purified	verified
Ad35.Mar(A)	G748-037	7	confirmed	CsCl purified	verified
Ad35.Ebo(SG)	G619-019A	9	confirmed	CsCl purified	verified
Ad35.Ebo(IC)	H539-037A	3	confirmed	CsCl purified	verified
Ad35.Mar(R)	H700-003A	6	confirmed	CsCl purified	verified
Ad26.Ebo(SG)	H539-074A	18	confirmed	CsCl purified	verified
Ad26.Ebo(Z)	H001-060A	11	confirmed	CsCl purified	verified
Ad26.Ebo(IC)	H539-085A	24	confirmed	CsCl purified	Verified
Ad26.Mar(A)	H129-067A	10	confirmed	CsCl purified	verified
Ad26.Mar(R)	H539-092A	12	confirmed	CsCl purified	verified
Ad35.empty	G849-053A	3	confirmed	CsCl purified	NA
Ad26.empty	G737-070A	26	confirmed	CsCl purified	NA

* Transgene integrity was verified by sequence analysis.

NA: Not applicable
