# Supplementary figures and images for: Ad35 and Ad26 Vaccine Vectors Induce Potent and Cross-Reactive Antibody and T-Cell Responses to Multiple Filovirus Species
Source: PLoS One. 2012 Dec 6;7(12):e44115. doi: 10.1371/journal.pone.0044115 (PMC3516506; doi:10.1371/journal.pone.0044115)

Supplemental Figure 1

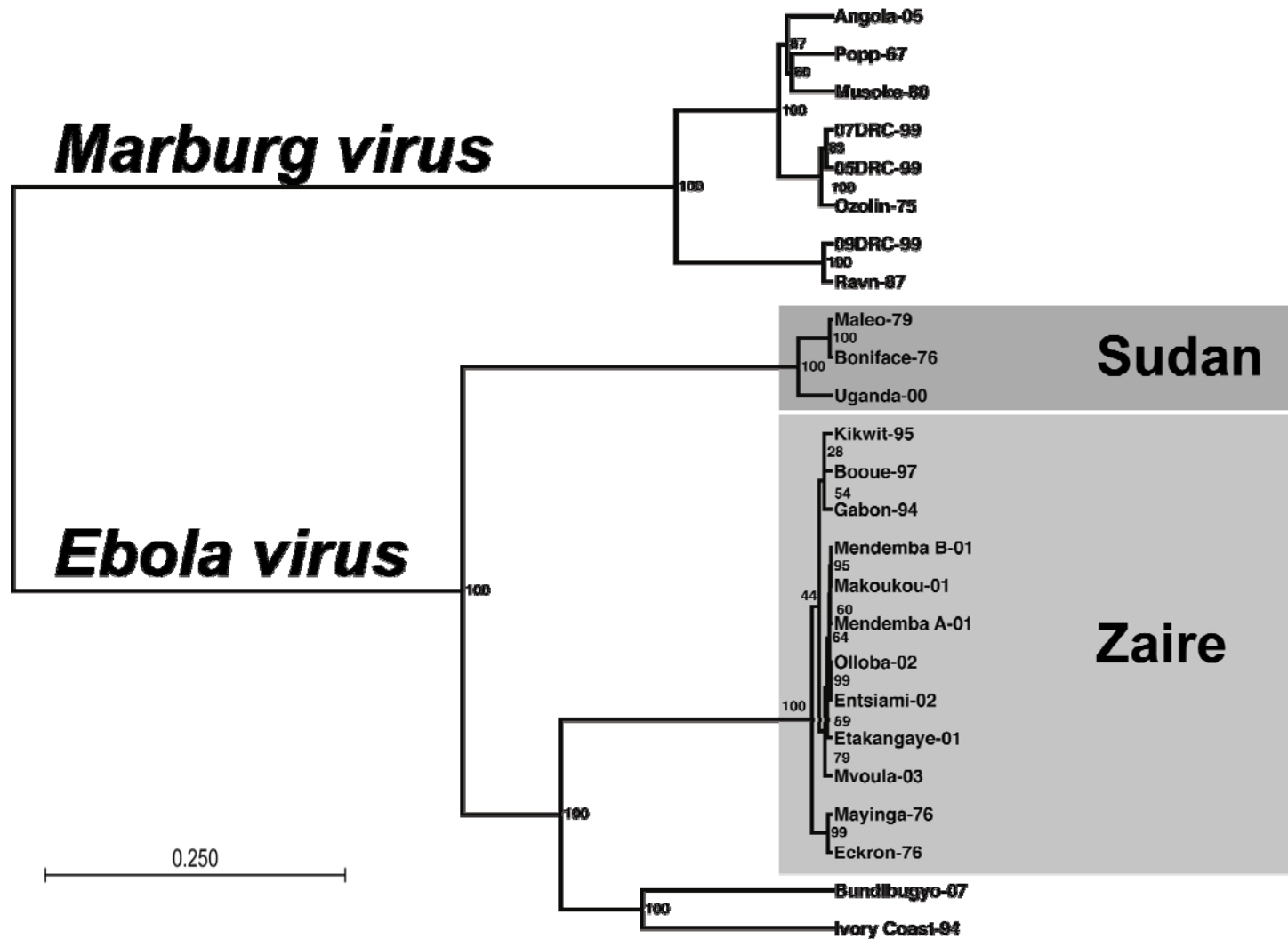

Supplement: Figure S1 — Phylogenetic relationship of the major filovirus strains that have caused outbreaks. Phylogenetic tree of the filovirus glycoprotein of all major filovirus strains using the UPGMA method. Confidence values are displayed at internal branches as percent of 1000 bootstraps. The branch length represents the phylogenetic distance. For better visualization the Zaire and Sudan strains are underlayed in grey. The tree was constructed with the Muscle algorithm for protein sequence alignment and the CLC workbench software for drawing of the tree. Accession numbers of the sequences used are described in material and methods. The numbers behind the strain names are the last two digits of year of strain isolation. Ebola Reston was not included as so far no human cases have been reported. (PDF) [file pone.0044115.s002.pdf]

## Supplemental Figure 2

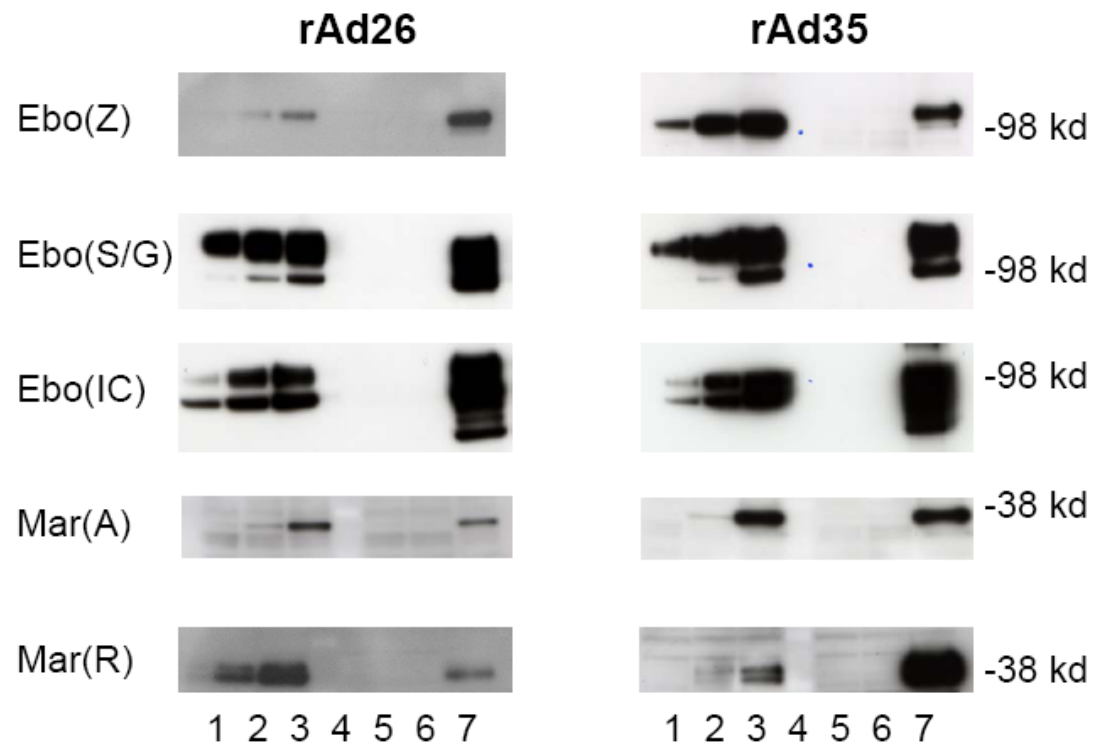

Supplement: Figure S2 — Filovirus glycoprotein expression by rAd26 and rAd35 vectors. Western blot analysis of A549 cells cultured to 70% confluency in 24-well plates infected with either rAd26 or rAd35 coding for either Ebola Zaire, Ebola Sudan/Gulu, Ebola Ivory Coast, Marburg Angola, or Marburg Ravn (lane 1–3 at an multiplicity of infection (MOI) of 10000, 25000, or 50000 for rAd26 and at an MOI of 1000, 2500, or 5000 for rAd35 vectors). MOI were based on vp/cell and in vitro transduction efficacy of Ad26 is lower than for Ad35 for A549 cells which was adjusted for by using a 10-fold higher MOI. The positive controls are cells infected with rAd5 (MOI 5000) coding for the same antigen (lane 7). The murine serum to detect the antigens is isolated out of Balb/c mice i.m. injected with rAd5 vectors four weeks before. The in this way generated Ebola specific sera were predominantly reactive against the GP1 (or/and GP0), whereas the Marburg specific sera specifically reacted with sera against GP2 in the western blot. Negative controls are untreated (lane 6) and rAd35.empty or rAd26.empty vector (lane 5, MOI of 50000 for rAd26 and MOI of 5000 for rAd35) infected A549 cells. Lane 4 is loaded with molecular weight marker. (PDF) [file pone.0044115.s003.pdf]
